# High-resolution ro-vibrational and rotational spectroscopy of HC_3_O^+^†

**DOI:** 10.1039/d3cp01976d

**Published:** 2023-07-04

**Authors:** Oskar Asvany, Sven Thorwirth, Philipp C. Schmid, Thomas Salomon, Stephan Schlemmer

**Affiliations:** a I. Physikalisches Institut, Universität zu Köln Zülpicher Str. 77 50937 Köln Germany asvany@ph1.uni-koeln.de

## Abstract

The ro-vibrational and pure rotational spectra of the linear ion HC_3_O^+^ have been investigated in a 4 K cryogenic ion trap instrument. For this, a novel action spectroscopic technique, called leak-out-spectroscopy (LOS, Schmid *et al.*, *J. Phys. Chem. A* 2022, **126**, 8111), has been utilized and characterized. In total, 45 ro-vibrational transitions within the fundamental band of the *ν*_1_ C–H stretching mode were measured with a band center at 3237.132 cm^−1^, as well as 34 lines from the combination band *ν*_2_ + *ν*_4_, and 41 lines tentatively identified as the combination band *ν*_2_ + *ν*_5_ + *ν*_7_, interleaved and resonant with *ν*_1_. Surprisingly, also two hot bands were detected despite the cryogenic operation temperature. Based on the novel action spectroscopy approach, a new double-resonance rotational measurement scheme was established, consisting of rotational excitation followed by vibrational excitation. Seven rotational transitions were observed between 89 and 180 GHz. Highly accurate spectroscopic parameters were extracted from a fit using all available data. In addition, a pulsed laser system has been employed to record a low resolution vibrational spectrum, in order to demonstrate the compatibility of such lasers with the LOS method.

## Abstract 2

Applying a 4 K cryogenic ion trap machine, the linear ion HC_3_O^+^ has been investigated by high-resolution ro-vibrational and pure rotational spectroscopy.

## Graphical abstract

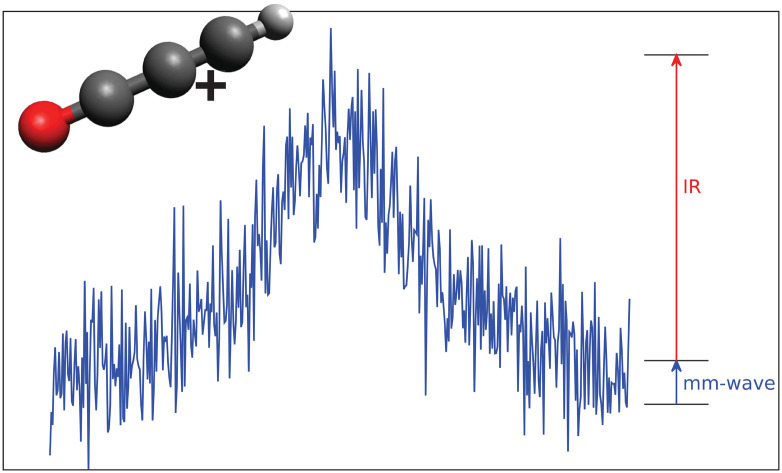

## 1 Introduction

To date, one of the very successful approaches for the investigation of molecular ions has been action spectroscopy, in which laser excitation is applied to a mass-selected ensemble of cold trapped ions. In contrast to other conventional spectroscopy methods, the absorption of a photon is not detected directly, but only its consequence in form of a change in ion signal. This makes action spectroscopy a highly sensitive method as counting of single ions can reach easily near 100% efficiency. Apart from this, such an approach yields unblended spectroscopic signals due to mass-selectivity and cryogenic operation. Some of the action spectroscopic methods widely applied today are photo-dissociation,^1,2^ also in the form of infrared multiple photon dissociation (IRMPD^3^) or the so-called messenger approach,^4–6^ laser induced reactions (LIR^7–9^), detachment of an electron from an anion,^10^ or laser induced inhibition of complex growth (LIICG^11–13^).

Once such action spectroscopic approaches are established, typically in form of ro-vibrational or rovibronic excitation, they can be extended to perform rotational spectroscopy by double resonance schemes. For this, a signal is generated by keeping the laser resonant on a transition (ro-vibrational or rovibronic), and this signal can then be modulated by a rotational transition connected with the quantum level probed by the laser. Examples of this rotational approach are double-resonance using LIR,^14–17^ photo-dissociation,^18,19^ LIICG,^20^ or the detachment of an electron^21,22^ and have been summarized recently in a review article by Asvany and Schlemmer.^23^

Very recently, a new action spectroscopic method has been introduced in our group, called leak-out-spectroscopy (LOS), and has been thoroughly tested and characterized *via* ro-vibrational excitation of C_3_H^+^.^24^ Tests on this and several other molecular cations and anions showed that LOS is generally applicable, operates at a wide range of temperatures, and produces spectra with a signal-to-noise ratio exceeding that of any action spectrum recorded in our laboratory by the aforementioned methods. In addition, LOS allows to analyze or even prepare the isomeric composition (be it structural or spin isomers) of the trapped ion ensemble.^25^

In this work, we enlarge the tool box of action spectroscopy, demonstrating high-resolution rotational spectroscopy *via* double-resonance applying LOS and recording first spectra employing a pulsed nanosecond OPO/OPA system. For this, we choose the linear molecular cation HC_3_O^+^. This molecule is the larger sibling of the astronomically well-known HCO^+^ ion,^26^ and has been recently characterized experimentally *via* low-resolution vibrational spectroscopy using Ne-tagging.^27^ More recently, it has also been detected in space based on two microwave lines measured in the laboratory.^28^ The methods outlined in this paper will enable the rotational spectroscopy of hitherto unexplored molecular ions, notably those of astrophysical interest.^29^

## 2 Experimental methods

The ro-vibrational and rotational transitions of HC_3_O^+^ were measured in the 4 K 22-pole ion trap instrument COLTRAP, which has previously been described in detail.^12,30^ In brief, the ions were generated in a storage ion source by electron ionization (*E*_e_ ≈ 24–30 eV) of suitable precursor mixtures. We applied dissociative ionization of propargyl alcohol vapour (H–C≡C–CH_2_OH, Sigma-Aldrich), which was admitted to the source *via* a slightly heated leakage valve, where it was diluted in He gas, or alternatively, we used a 1 : 1 : 1 mixture of C_2_H_2_, CO and He. Every second, a pulse of several ten thousand mass-selected HC_3_O^+^ ions (*m* = 53 u) was injected into the 22-pole ion trap, cooled by a Sumitomo coldhead to cryogenic temperatures and filled with low density Ne gas. Operation above the freeze-out temperature of Ne, with the trap held at about *T* = 12 K, allowed to introduce the Ne gas in a continuous fashion (number density on the order of ∼10^13^ cm^−3^), whereas for the operation at 4 K a 1 : 3 Ne : He gas mixture was pulsed into the trap (alternatively, an Ar : He mixture was also tested successfully).

Once trapped, the ro-vibrational transitions of HC_3_O^+^ were detected using the novel LOS method.^24^ This method is based on the escape of a trapped ion after collision-induced transfer of vibrational to kinetic energy: after a cooling period of about 40 ms in the ion trap, the ions were irradiated for several 100 ms by an IR beam traversing the trap. In case of the laser being resonant with a ro-vibrational transition, vibrationally excited HC_3_O^+^ ions could be quenched by collisions with the noble gas atoms present in the trap. Meeting a Ne atom, *e.g.*, the neutral-to-ion mass ratio of 20 : 53 allows to transfer a substantial part of the vibrational energy into kinetic energy of the ion, namely a maximum of 20/(20 + 53) × 3200 cm^−1^ = 877 cm^−1^ ≈ 0.11 eV. A similar calculation for Ar, with a more favourable mass ratio, yields about 0.17 eV maximum kinetic energy, albeit with the drawback of a higher freeze-out temperature of Ar (about 40 K). By keeping the electrostatic barrier at the exit side of the trap well below 110 mV (in the case of Ne), the accelerated ions may escape in that direction, and fly towards the ion detector where they are finally counted, while the non-excited, thermal ions are kept in the cold trap. By repeating these trapping cycles at 1 Hz and counting the escaping HC_3_O^+^ ions as a function of the laser wavenumber, a (ro)vibrational spectrum can be recorded.

In this work, we used two different IR sources operating in the 3 μm spectral region, a low-resolution pulsed one, and a continuous wave (cw) high-resolution one. The IR beam entered the vacuum environment of COLTRAP *via* a 0.6 mm thick diamond window (Diamond Materials GmbH), traversed the 22-pole trap, exited the vacuum system *via* a CaF window, after which it was stopped by a power meter. The pulsed IR radiation is produced by a table-top LaserVision optical parametric oscillator/amplifier (OPO/OPA) system. The OPO/OPA system is pumped with a 1064 nm Nd:YAG laser (Continuum Surelite-Ex) operating at 10 Hz and with maximum pulse energies of 600 mJ and a typical duration of several nanoseconds. The pump laser was operated in unseeded mode. The IR laser wavelength is monitored with a wavemeter (HighFinesse WS-5) with a manufacturer-stated accuracy of 0.1 cm^−1^. Typical IR pulse energies in the spectral range investigated here are on the order of 2–5 mJ (measured at the exit of the trap experiment). The high-resolution measurements, on the other side, were carried out with a cw OPO (Aculight Argos Model 2400, Model C). The power was on the order of 200 mW. The irradiation time was controlled by a laser shutter (model Thorlabs SH05). The frequency of the IR radiation has been measured by a wavemeter (Bristol model 621A) with an accuracy reaching 0.001 cm^−1^ in well-adjusted settings. Some more comments about the accuracy and precision of the high-resolution IR experiments are given in the ESI.†

For detecting pure rotational transitions, we used a double resonance scheme involving LOS (see Section 5 for details). To generate the necessary mm-wave radiation, a rubidium-clock-referenced microwave synthesizer (Rohde & Schwarz SMF 100A) driving an amplifier-multiplier chain (Virgina Diodes Inc. WR9.0M-AMC) was employed. The radiation was focused by an ellipsoidal mirror (*f* = 43.7 mm^15^) before entering the vacuum environment through the diamond window. Both the IR and mm-wave radiation sources were used simultaneously, and their beams combined by a small hole in the ellipsoidal mirror through which the narrow IR beam could pass.

## 3 Quantum-chemical calculations

High-level CCSD(T) quantum-chemical calculations of HC_3_O^+^ and its isoelectronic sibling HC_3_N were performed with the CFOUR program^32,33^ and have already been described in our earlier report on the low-resolution infrared spectroscopic detection.^27^ This paper should be consulted for any technical details regarding the theoretical methods. Complementary to the wavenumber estimates of the vibrational fundamentals of HC_3_O^+^ given there, information of molecular parameters of relevance for an analysis of high-resolution spectra are given in Table 1. As can be seen, for HC_3_N a comparison of calculated and experimental rotation–vibration interaction (*α*_*i*_) as well as ℓ-type doubling (*q*_*i*_) parameters shows very good agreement throughout, suggesting that the fc-CCSD(T)/cc-pVTZ level of theory provides a good description of the molecular force field. Owing to isoelectronicity, it is not entirely surprising that corresponding calculations of HC_3_O^+^ yield very similar results that are deemed useful for an analysis of high-resolution (infrared) spectroscopic data of this molecular ion such as those presented in the following.

**Table 1:** Calculated (fc-CCSD(T)/cc-pVTZ) and experimental rotation–vibration interaction (*α*_*i*_) as well as ℓ-type doubling (*q*_*i*_) constants of the fundamental vibrational modes *ν*_*i*_ of HC_3_N and HC_3_O^+^ (in MHz). The fundamental vibrational wavenumbers of HC_3_O^+^ are given in cm^−1^

Mode	HC_3_N				HC_3_O^+^		
	*α*_*i*,calc_	*α*_*i*,exp_ a	*q*_*i*,calc_	*q*_*i*,exp_ a	*α*_*i*,calc_	*q*_*i*,calc_	*ν*_*i*,BE_ b
*ν*_1_ C–H stretch	7.06	7.28	—	—	7.31	—	3231
*ν*_2_ C–O/N stretch	21.93	21.57	—	—	22.84	—	2316
*ν*_3_ C–C stretch	13.87	13.94	—	—	14.56	—	2074
*ν*_4_ C–C stretch	10.65	10.96	—	—	8.94	—	911
*ν*_5_ C–C–H bending	−1.68	−1.57	2.42	2.54	−0.49	2.13	773
*ν*_6_ C–C–O/N bending	−9.07	−9.24	3.48	3.58	−8.00	3.06	558
*ν*_7_ C–C–C bending	−13.80	−14.47	6.25	6.54	−18.93	7.39	169

a see Tamassia *et al.*^31^

b Best estimate value, see Thorwirth *et al.*^27^

## 4 Infrared results and discussion

Like isoelectronic cyanoacetylene, HC_3_N, the HC_3_O^+^ molecular ion is a linear molecule with a ^1^Σ electronic ground state. The only previous laboratory spectroscopic investigations of this molecular ion are that of the neon-tagged variant, providing an overview of the vibrational spectrum^27^ by using the widely tunable FELIX light source^35^ and a pulsed OPO, subsequently followed by measurement of two rotational transitions of the bare ion by Fourier transform microwave spectroscopy,^28^ leading to the detection of four rotational lines of HC_3_O^+^ in the molecular cloud TMC-1. The present work summarizes the first high-resolution ro-vibrational investigation of HC_3_O^+^, originally targeted at the fundamental C–H stretching mode *ν*_1_. As it also involves a new spectroscopic method that has been employed to measure the pure rotational spectrum, our approach is described in some more detail in the following.

### 4.1 Measurements applying LIICG at 4 K

Based on the low-resolution infrared spectroscopic detection,^27^ first ro-vibrational lines assigned to the *ν*_1_ band were detected with the LIICG method (mentioned in the introduction) in the summer of 2020. The band was found centered at 3237.13 cm^−1^. As the newly developed LOS-method is close-to background-free and thus features a much superior signal-to-noise ratio, the early measurements were repeated and greatly extended using LOS, and the LIICG results are only shown as part of the ESI† for comparison.

### 4.2 LOS-measurements at low spectral resolution

**Fig. 1:**
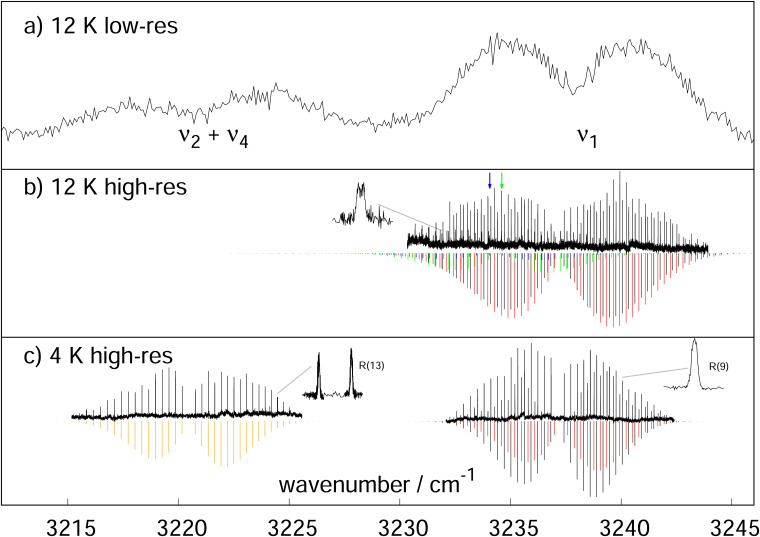
LOS-spectra of HC_3_O^+^ using Neon as collision partner in the region of the C–H stretching mode *ν*_1_, recorded at the given nominal trap temperatures. (a) Spectrum using a pulsed OPO/OPA system. As the laser was not seeded, its resolution is on the order of 1 cm^−1^. The envelopes of the *P*- and *R*-branches of the bands *ν*_1_ and the combination band *ν*_2_ + *ν*_4_ are clearly discernible. (b) High-resolution measurement using a cw OPO. In addition to the *ν*_1_ band (PGOPHER^34^ simulation at 25 K given as black sticks), we could also identify two additional hot bands (simulated with green and blue sticks), as well as a band tentatively assigned as *ν*_2_ + *ν*_5_ + *ν*_7_ (simulated with red sticks). The surprising appearance of the two hot bands is discussed in the text. One hot band features a doublet structure (see inset). Two weak *Q*-branches of these hot bands could be identified, whose positions are indicated by arrows. (c) By pulsing a 3 : 1 He : Ne mixture into the ion trap, the experiment could be further cooled down to 4 K, so that the hot bands finally are frozen out. The stick spectra (black, red, orange) have been simulated at a rotational temperature of 12 K. The inset on the right shows a zoom of the measurement for the *R*(9) transition of *ν*_1_, exhibiting a slightly saturated Doppler profile with FWHM of ∼40 MHz, and yielding a kinetic temperature of HC_3_O^+^ of about 12 K. The left inset shows the split nature of the *R*(13) transition in the *ν*_2_ + *ν*_4_ band, pointing towards a perturbation in the *J* = 14 level of the upper vibrational state.

A LOS-measurement at low spectral resolution, applying the pulsed OPO/OPA system, is shown in panel (a) of Fig. 1. For this measurement the 22-pole ion trap was operated at a temperature of 12 K to avoid the freeze-out of Neon. In an attempt to possibly collect information about the nature of the second vibrational band observed in the initial infrared study^27^ of neon-tagged HC_3_O^+^, a survey scan was performed in the range from 3204 to 3278 cm^−1^. Indeed, two vibrational bands were observed, a stronger one located at 3237 cm^−1^ and a somewhat weaker at 3221 cm^−1^. As the linewidth of the unseeded laser was about 1 cm^−1^, individual ro-vibrational lines were not resolved but the envelopes of the *P*- and *R*-branches of both bands are clearly discernible. The separation of some 16 cm^−1^, is the same as the one found for the neon-tagged variant suggesting that actually both bands stem from the bare ion and neither one is a consequence of rare gas tagging. Consequently, from these new measurements the earlier spectroscopic assignment^27^ is calling for revision, *i.e.*, the stronger band at 3237 cm^−1^ is now identified as *ν*_1_, and hence found blue-shifted by 6 cm^−1^ from the theoretical best estimate value (see Table 1). The somewhat weaker band is finally identified as the *ν*_2_ + *ν*_4_ combination, in good agreement with our anharmonic force field calculations and also with a value of *ν*_2_ + *ν*_4_ = 3219 cm^−1^ obtained from the measured fundamental vibrational wavenumbers derived in our study with FELIX.^27^ As the Ne-induced wavenumber shifts of the vibrational bands of HC_3_O^+^ have been calculated to be generally very small and also to act to the red for *ν*_1_,^27^ we attribute the significant wavenumber mismatch between the earlier IRPD-study of Ne-tagged HC_3_O^+^ (leading to our erroneous identification of the lower wavenumber band as *ν*_1_) and the new LOS measurements presented here to poor wavenumber calibration in the former measurement. A graphical comparison of both measurements is given in the ESI.†

We want to stress here the importance of the successful demonstration of the LOS method in combination with a pulsed laser source. For instance, the LIICG method mentioned above is known to have difficulties in a pulsed irradiation mode. In that method the inhibition of complex formation with He due to the excitation of the ion of interest is observed. It is obvious that this signal suffers from the very poor duty cycle of a pulsed laser because complex formation is only reduced during the very short time of irradiation. In contrast, in LOS the signal only depends on the number of excited ions. This number can be similar comparing pulsed and continuous radiation as already discussed for the laser induced reaction (LIR) approach.^8^ Therefore, a LOS signal was expected also for the pulsed irradiation. However, early non-synchronized attempts to measure the low-resolution spectrum with LOS suffered from excessive background counts. In order to substantially reduce these background counts, the kicked-out ions have been counted only in a small time window of ∼5 ms width directly after each laser excitation pulse. For a total trapping time of 2.7 s this corresponds to the signal of about 26 laser shots. The named window opening time matches typical collision and flight times of the ions. For the correct timing, the whole trapping cycle is synchronized to the pulsed OPO/OPA system using a pulse generator (Quantum Composers Model 9514).

### 4.3 LOS-measurements at high spectral resolution

First high-resolution LOS measurements of the *ν*_1_ mode were performed with the cw OPO system and at a trap temperature of 12 K, the results of which are shown in panel (b) of Fig. 1. The narrow linewidth of this OPO (≲1 MHz) allows to fully resolve individual ro-vibrational transitions and also to determine reliably the kinetic temperature of HC_3_O^+^ from the line profiles observed in the ion trap. Very surprisingly, the range from 3230 to 3244 cm^−1^ features not one single but at least four ro-vibrational bands, one of which is expected to be the *ν*_1_ vibrational fundamental. A second band interleaved with *ν*_1_ is of comparable intensity and also exhibits the structure reminiscent of a Σ–Σ band (simulated with red sticks in Fig. 1). The two remaining and weaker bands are clearly identified as hot bands originating from energetically low-lying bending modes (indicated as blue and green sticks in Fig. 1b). Support for this assignment is provided by the presence of weak *Q*-branches (their positions are indicated by blue and green arrows) and through partially resolved ℓ-type doubling in at least one of these bands for higher values of *J* (see inset in panel (b)). The appearance of hot bands may seem astonishing, as the ions are stored in a 12 K ion trap though apparently with some heating effects.^36^ The analysis of the spectrum gives a rotational temperature of about 25 K and a kinetic temperature of about 30 K. The lowest fundamental mode of HC_3_O^+^, however, the C–C–C bending vibration *ν*_7_, is calculated to be much higher in energy, at about 169 cm^−1^ (≅243 K),^27^ and thus should have a small probability to be excited even at these temperatures of the experiment. A similar observation of hot bands has been made for the linear molecule C_3_H^+^ in our recent paper introducing the LOS method.^24^ As such hot bands have never been observed before using other action spectroscopic methods operating at cryogenic trap temperatures, our findings seem to indicate some excitation mechanism specific to the LOS method. A more detailed discussion can be found at the end of this paper.

### 4.4 LOS-measurements at high resolution and 4 K

Subsequently, we were able to record spectra at a trap temperature of 4 K, *i.e.*, well below the freeze-out temperature of Neon. For this, the trap was filled continuously with He (at a number density of ∼10^13^ cm^−3^) in order to effectively catch the ions arriving from the ion source, and additionally a 3 : 1 He : Ne mixture was pulsed into the trap. With these conditions, two band regions were scanned: the *ν*_1_ band range from 3232 cm^−1^ to 3242 cm^−1^ as well as the region from 3215 cm^−1^ to 3226 cm^−1^ featuring the *ν*_2_ + *ν*_4_ combination band regime (see panel c in Fig. 1).

At this temperature, the *ν*_1_ region looks comparably tidy. The two designated hot bands visible in panel (b) have vanished lending additional support to their assignment as hot bands. The fact that the two remaining bands are still observed at 4 K may be viewed as an indication that both bands originate from the ground vibrational state. The dominating Σ–Σ type band is finally identified as the *ν*_1_ fundamental. For this band and at this temperature, 33 lines are assigned which can be further complemented to a total of 45 with lines detected in the LOS spectrum observed at 12 K (Fig. 1, panel b). It should be mentioned that these ro-vibrational lines belong to the same band initially detected with the LIICG method. The finally derived transition wavenumbers of this band are summarized in Table 2. The transitions feature narrow Doppler widths of ∼40 MHz FWHM (see zoom of *R*(9) line in panel (c)). Some lines seem slightly saturated, and a fit using a saturated Doppler profile yields a kinetic temperature around 12 K, again somewhat hotter than the nominal trap temperature. Also, the simulation of the ro-vibrational line intensities with PGOPHER,^34^ shown as black sticks for the *ν*_1_ band, is compatible with a rotational temperature on the order of 12 K. An effective fit of the data collected in Table 2 yields the rotational constants *B*_0_ = 4460.92(7) MHz and *B*_1_ = 4447.27(7) MHz with the band center located at *ν*_1_ = 3237.13203(9) cm^−1^.

**Table 2:** Assignments, wavenumbers (in cm^−1^) and fit residuals of 45 ro-vibrational transitions in the *ν*_1_ band of HC_3_O^+^. Data is based on 12 K measurement seen in Fig. 1b. The accuracy of the used wavemeter is a few 1 × 10^−3^ cm^−1^

*J*′ ← *J*′′	Experimental	Obs–calc
21 ← 22	3230.3765	0.0011
20 ← 21	3230.6926	0.0006
19 ← 20	3231.0080	0.0002
18 ← 19	3231.3224	−0.0003
17 ← 18	3231.6364	−0.0001
16 ← 17	3231.9492	−0.0003
15 ← 16	3232.2617	0.0001
14 ← 15	3232.5728	0.0000
13 ← 14	3232.8830	0.0000
12 ← 13	3233.1927	0.0003
11 ← 12	3233.5012	0.0003
10 ← 11	3233.8083	−0.0001
9 ← 10	3234.1150	−0.0001
8 ← 9	3234.4211	0.0002
7 ← 8	3234.7258	0.0001
6 ← 7	3235.0299	0.0003
5 ← 6	3235.3331	0.0004
4 ← 5	3235.6353	0.0005
3 ← 4	3235.9361	0.0001
2 ← 3	3236.2361	−0.0002
1 ← 2	3236.5359	0.0002
0 ← 1	3236.8345	0.0003

1 ← 0	3237.4276	−0.0009
2 ← 1	3237.7235	−0.0007
3 ← 2	3238.0185	−0.0005
4 ← 3	3238.3128	−0.0001
5 ← 4	3238.6062	0.0002
6 ← 5	3238.8980	−0.0001
7 ← 6	3239.1899	0.0006
8 ← 7	3239.4800	0.0004
9 ← 8	3239.7693	0.0003
10 ← 9	3240.0577	0.0003
11 ← 10	3240.3439	−0.0011
12 ← 11	3240.6308	−0.0009
13 ← 12	3240.9165	−0.0008
14 ← 13	3241.2013	−0.0008
15 ← 14	3241.4859	−0.0001
16 ← 15	3241.7687	−0.0003
17 ← 16	3242.0510	−0.0001
18 ← 17	3242.3322	0.0001
19 ← 18	3242.6124	0.0001
20 ← 19	3242.8922	0.0005
21 ← 20	3243.1696	−0.0004
22 ← 21	3243.4469	−0.0006
23 ← 22	3243.7236	−0.0003

The ground state rotational constant *B*_0_ is found in very good agreement with the previous value obtained in the microwave region.^28^ Interestingly, the rotation–vibration interaction constant *α*_1_ = 13.65(10) MHz derived from this analysis is only qualitatively consistent with the calculated value provided in Table 1 (7.31 MHz) and off by about a factor of two.

The transition wavenumbers of the unexpected (apparent) Σ–Σ-type band associated with *ν*_1_ are collected in Table 3. As indicated there, the very low-*J* transitions *P*(1) and *R*(0) could not be detected, and additionally, the *P*(3) and *R*(1) lines are split into doublets (separated by some 0.006 cm^−1^), most likely due to a perturbation in the upper *J* = 2 state. An effective fit of all unsplit data yields the rotational constants *B*_0_ = 4460.68(12) MHz and *B*_*v*_ = 4448.49(12) MHz (*ν* = 3237.00361(18) cm^−1^). Again, the ground state rotational constant is in good agreement with the microwave value. Unfortunately, spectroscopic assignment of the upper state is not straightforward as no binary combination modes other than *ν*_2_ + *ν*_4_ are expected to be present in the vicinity of *ν*_1_. Closer inspection of the CCSD(T) anharmonic force field calculations reveals one ternary combination band *ν*_2_ + *ν*_5_ + *ν*_7_ to be located slightly higher in wavenumber, by some 2 cm^−1^ only. The upper state of this mode is composed of the *ν*_2_ stretching mode as well as the *ν*_5_ and *ν*_7_ bending modes, hence featuring Σ^±^ and Δ subbands. Conceivably, the Σ^+^ subband of the *v*_2_ + *v*_5_ + *v*_7_ state and the *v*_1_ state form a Fermi resonance system with the *ν*_2_ + *ν*_5_ + *ν*_7_ band also borrowing intensity from the *ν*_1_ fundamental band. Even the *ν*_2_ + *ν*_4_ band may be part of this resonance system. While a quantitative description of the perturbation problem based on the experimental data is not feasible, a simple second order de-perturbation approach is in support of this working assumption and provided as part of the ESI.†

**Table 3:** Assignments, wavenumbers (in cm^−1^) and fit residuals of 41 ro-vibrational transitions in the *ν*_2_ + *ν*_5_ + *ν*_7_ combination band of HC_3_O^+^. Data is based on 12 K measurement seen in Fig. 1b. The accuracy of the used wavemeter is a few 1 × 10^−3^ cm^−1^

*J*′ ← *J*′′	Experimental	Obs–calc
20 ← 21	3230.8820	0.0006
19 ← 20	3231.1952	−0.0002
18 ← 19	3231.5084	−0.0001
17 ← 18	3231.8208	0.0001
16 ← 17	3232.1320	−0.0002
15 ← 16	3232.4428	0.0001
14 ← 15	3232.7526	0.0001
13 ← 14	3233.0614	0.0001
12 ← 13	3233.3697	0.0003
11 ← 12	3233.6770	0.0003
10 ← 11	3233.9833	0.0002
9 ← 10	3234.2886	−0.0001
8 ← 9	3234.5934	−0.0001
7 ← 8	3234.8975	0.0001
6 ← 7	3235.2008	0.0002
5 ← 6	3235.5033	0.0003
4 ← 5	3235.8045	−0.0001
3 ← 4	3236.1049	−0.0004
2 ← 3	3236.4020/3236.4083	Doublet
1 ← 2	3236.7047	0.0002

2 ← 1	3237.8900/3237.8961	Doublet
3 ← 2	3238.1879	−0.0005
4 ← 3	3238.4830	0.0002
5 ← 4	3238.7761	−0.0002
6 ← 5	3239.0695	0.0004
7 ← 6	3239.3616	0.0005
8 ← 7	3239.6525	0.0003
9 ← 8	3239.9430	0.0004
10 ← 9	3240.2322	0.0001
11 ← 10	3240.5199	−0.0009
12 ← 11	3240.8080	−0.0006
13 ← 12	3241.0948	−0.0008
14 ← 13	3241.3810	−0.0008
15 ← 14	3241.6672	0.0000
16 ← 15	3241.9516	0.0000
17 ← 16	3242.2355	0.0003
18 ← 17	3242.5185	0.0005
19 ← 18	3242.8003	0.0004
20 ← 19	3243.0811	0.0002
21 ← 20	3243.3603	−0.0007
22 ← 21	3243.6401	−0.0001

Concerning the *ν*_2_ + *ν*_4_ combination band, in total 34 lines of this band located at 3220.5 cm^−1^ were measured, the transition wavenumbers of which are collected in Table 4. A simulation of its ro-vibrational lines is depicted as orange sticks in panel c of Fig. 1. Interestingly, the *P*(15) and *R*(13) lines of this band appear as doublets split by about 0.016 cm^−1^ (the latter shown as a zoom in panel c of Fig. 1), pointing towards a perturbation in the *J* = 14 state of *ν*_2_ + *ν*_4_. Also, at least three additional weak lines have been detected in the *R*-branch whose identity has not been clarified yet. An effective fit of the unsplit lines in Table 4 yields the rotational parameters *B*_0_ = 4460.57(13) MHz and *B*_2+4_ = 4437.19(13) MHz (*ν*_2+4_ = 3220.46732(12) cm^−1^). Here, the effective rotation–vibration interaction parameter *α*_2+4_ = 23.38(18) MHz is qualitatively consistent with 31.78 MHz estimated from the sum of *α*_2_ and *α*_4_ given in Table 1.

**Table 4:** Assignments, wavenumbers (in cm^−1^) and fit residuals of 34 ro-vibrational transitions in the *ν*_2_ + *ν*_4_ combination band of HC_3_O^+^. Data is based on 4 K measurement seen in Fig. 1c. The accuracy of the used wavemeter is a few 1 × 10^−3^ cm^−1^

*J*′ ← *J*′′	Experimental	Obs–calc
16 ← 17	3215.1959	0.0001
15 ← 16	3215.5178	*
14 ← 15	3215.8331/3215.8493	Doublet
13 ← 14	3216.1600	0.0006
12 ← 13	3216.4773	−0.0001
11 ← 12	3216.7935	−0.0002
10 ← 11	3217.1083	0.0000
9 ← 10	3217.4213	−0.0002
8 ← 9	3217.7330	0.0000
7 ← 8	3218.0429	−0.0001
6 ← 7	3218.3513	−0.0001
5 ← 6	3218.6582	0.0000
4 ← 5	3218.9636	0.0000
3 ← 4	3219.2673	0.0000
2 ← 3	3219.5694	0.0000
1 ← 2	3219.8702	0.0001
0 ← 1	3220.1694	0.0002

1 ← 0	3220.7630	0.0001
2 ← 1	3221.0576	0.0003
3 ← 2	3221.3504	0.0001
4 ← 3	3221.6419	0.0002
5 ← 4	3221.9313	−0.0003
6 ← 5	3222.2195	−0.0003
7 ← 6	3222.5062	−0.0004
8 ← 7	3222.7917	0.0000
9 ← 8	3223.0751	−0.0003
10 ← 9	3223.3573	−0.0001
11 ← 10	3223.6379	0.0002
12 ← 11	3223.9168	0.0003
13 ← 12	3224.1941	0.0004
14 ← 13	3224.4623/3224.4786	Doublet
15 ← 14	3224.7420	*
16 ← 15	3225.0150	−0.0004
17 ← 16	3225.2856	−0.0003

Finally, the spectroscopic assignment of the vibrational hot bands seen in panel b of Fig. 1 proved challenging, too. These hot bands could originate from the lowest lying C–C–C bending vibration *ν*_7_, its first overtone 2*ν*_7_, or the C–C–O bending motion *ν*_6_. A detailed discussion about their tentative assignment and a list of transition frequencies is given in the ESI.†

## 5 Pure rotational measurements

**Fig. 2:**
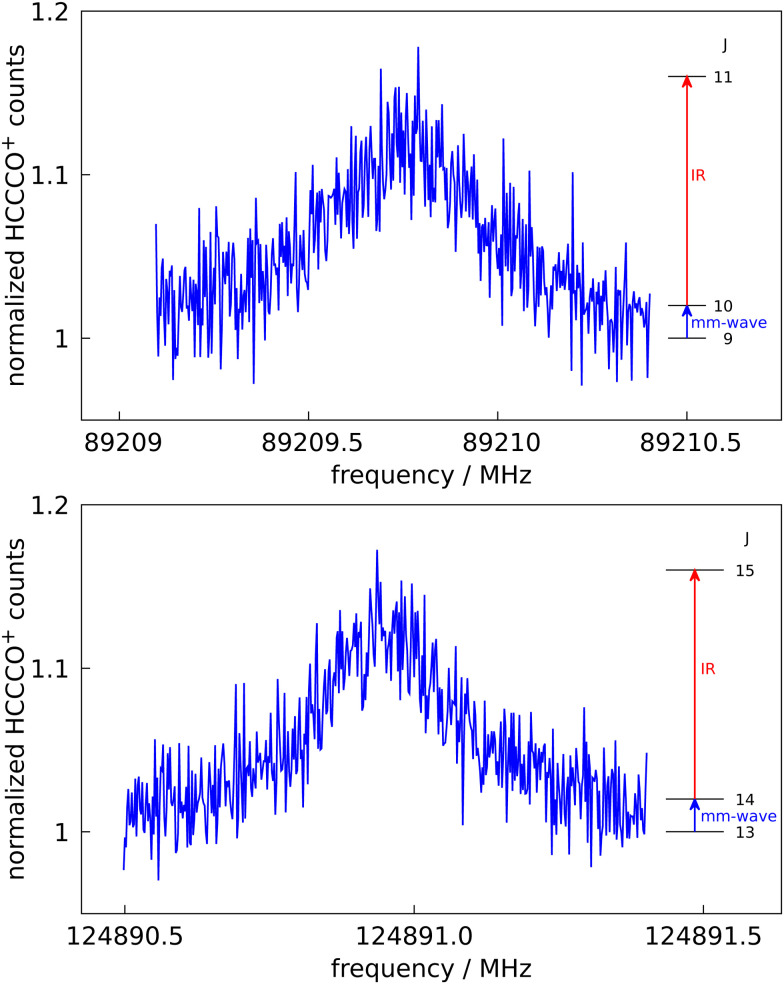
Pure rotational transitions *J* = 10 ← 9 and *J* = 14 ← 13 of HC_3_O^+^ measured by a double resonance scheme, in which the mm-wave excitation (blue arrows in insets) is followed by IR excitation (red arrows in insets) into the *ν*_1_ vibrational band and subsequent leak-out from the trap. The 89 GHz line has a Lorentzian shape due to power broadening effects. The step sizes are 3 kHz and 2 kHz, respectively. The data signal counts are normalized and therefore the baselines are unity.

The last section and our previous work^24^ have demonstrated that ro-vibrational spectroscopy can be performed using LOS with high signal-to-noise ratio. Having such a novel action spectroscopic method available for ro-vibrational spectroscopy, it is natural to extend such a scheme into the domain of pure rotational spectroscopy *via* double resonance, as has been demonstrated in our group by many examples.^14–20,23^ The main purpose of such studies is to determine the rotational constants and other spectroscopy parameters with much higher accuracy than by the ro-vibrational spectroscopy as presented here, thus allowing for an unambiguous search for those molecules in radio astronomical observations. In such a double resonance scheme, the IR and mm-wave radiation are applied simultaneously to the ions. The frequency of the IR photon is kept fixed at a ro-vibrational transition starting from a rotational level of the vibrational ground state, resulting in a detectable and constant LOS signal. The mm-wave photon then excites a rotational transition starting or ending on the rotational quantum state probed by the IR laser, thus decreasing or increasing the LOS signal. A rotational line can therefore be recorded by modulating this LOS-signal, *i.e.*, by scanning the frequency of the mm-wave source as shown by the examples in Fig. 2.

Such spectra were recorded in individual measurements in which the mm-wave frequency (blue arrows in Fig. 2) was stepped in an up-and-down manner several times. The frequency steps were kept constant in individual experiments, and varied between 2 and 10 kHz. One difficulty in these experiments were the very narrow IR transitions (with FWHM of about 40 MHz at 4 K) on which the IR source (red arrows in Fig. 2) had to be stabilized. Due to this challenge and other drifting experimental conditions, the spectroscopic data were normalized employing a frequency switching procedure, *i.e.*, by dividing the HCCCO^+^ counts monitored while scanning the spectral window of interest by the counts at an off-resonant mm-wave reference frequency outside this window. Therefore, the baselines in Fig. 2 are close to unity. The on-resonance signal enhancements are of order 12%. Transition frequencies were determined by adjusting the parameters of an appropriate line shape function to the experimental spectrum in a least-squares procedure. In case of excessive mm-wave power the power broadening effect leads to a line shape being very close to a Lorentzian as discernible for the 89 GHz line in Fig. 2. This power broadening occurred mainly in the low-frequency lines where the power output of our mm-wave source was ample.

Using the *ν*_1_ IR band for the double resonance, in total seven rotational lines have been detected which are summarized in Table 5. Every line has been measured several times (typically at least 10 times) and the combined values for the frequency position and uncertainty are given there. Also given in Table 5 are available literature values from Cernicharo *et al.*,^28^ which show very good agreement with the measurements of this work.

**Table 5:** Rotational transition frequencies of HC_3_O^+^. The frequencies are given in MHz, and the uncertainties and obs–calc values in kHz

*J*′ ← *J*′′	Experimental	unc	obs–calc	Comment
2 ← 1	17 842.3387	5.0	1.0	Ref. 28
3 ← 2	26 763.4749	5.0	−1.5	Ref. 28
4 ← 3	35 684.590	10.0	11.2	Ref. 28
5 ← 4	44 605.648	10.0	15.1	Ref. 28
10 ← 9	89 209.7533	1.8	−2.4	4 K, this work
11 ← 10	98 130.2691	2.3	3.0	4 K, this work
12 ← 11	107 050.6415	2.4	−2.2	4 K, this work
13 ← 12	115 970.8725	2.4	−3.8	4 K, this work
14 ← 13	124 890.9548	1.6	3.0	4 K, this work
19 ← 18	169 488.5486	3.1	−2.4	13 K, this work
20 ← 19	178 407.4573	20.0	26.8	17 K, this work

One advantage of the double resonance approach is that it can check the connectivity of quantum levels. In order to corroborate the identification and assignment of the *ν*_2_ + *ν*_5_ + *ν*_7_ combination band observed at 4 K interleaved with the *ν*_1_ (see Fig. 1 panel c), we performed a double resonance rotational experiment, consisting of the rotational excitation *J* = 10 ← 9 in the ground state, followed by the IR transition *J* = 11 ← 10 at 3240.5199 cm^−1^ in the *R*-branch of this band. We could detect a signal similar to those shown in Fig. 2, albeit with a poorer S/N ratio, thus confirming the *ν*_2_ + *ν*_5_ + *ν*_7_ combination band to originate from the vibrational ground state, and thus excluding it to be a hot band as the other two bands detected at 12 K. Also, the assignment of the applied ro-vibrational transition to *J* = 11 ← 10 as given in Table 3 is thus confirmed.

## 6 Spectroscopic parameters

The rotational and ro-vibrational data from the preceding sections were used for the determination of the spectroscopic parameters of all observed bands. Due to the very high accuracy of the pure rotational measurements (these are more than 3 orders of magnitude more accurate than the IR data), these were fit first, obtaining a good rms value of the weighted obs–calc values of 1.214 (see the corresponding obs–calc values in Table 5). We included only the rotational parameters *B* and *D* in the fit, while inclusion of the sextic distortion constant *H* was not meaningful. The rotational constants were then kept fixed for the fit of those ro-vibrational bands which are connected to the ground state. The corresponding spectroscopic parameters are summarized in Table 6. The resulting obs–calc values for the ro-vibrational fits are given in Tables 2–4. These are typically a few 0.0001 cm^−1^ and indeed confirm the good quality of the measurements. For instance, the average obs–calc deviation of the measured lines of the *ν*_1_ band is only 0.0004 cm^−1^. The spectroscopic parameters of the two hot bands are given in Table 7 with the corresponding line lists and obs–calc values given in the ESI.†

**Table 6:** The best fit spectroscopic parameters of HC_3_O^+^ are obtained by fitting the data given in Tables 2–5 with the program PGOPHER.^34^ The numbers in parentheses give the uncertainty of the last digits. The ground state was fixed to the mm-wave data

Parameter	Ground	*ν*_1_	*ν*_2_ + *ν*_4_	*ν*_2_ + *ν*_5_ + *ν*_7_	Unit
*ν*		3237.1318(1)	3220.4668(1)	3237.3005(2)	cm^−1^
*B*	4460.58846(11)	4446.96(4)	4437.52(5)	4448.62(5)	MHz
*D*	0.5034(3)	0.53(8)	1.8(2)	1.0(1)	kHz

**Table 7:** The best fit spectroscopic parameters of HC_3_O^+^ for the two hot bands observed in Fig. 1(b). These two bands are tentatively assigned to *ν*_1_ + *ν*_7_ ← *ν*_7_ (^1^Π ← ^1^Π), *ν*_1_+ 2*ν*_7_ ← 2*ν*_7_ (^1^Δ ← ^1^Δ), or *ν*_1_ + *ν*_6_ ← *ν*_6_ (^1^Π ← ^1^Π), with *ν*_7_ and *ν*_6_ being the lowest bending modes of this molecule. A detailed discussion about assignment suggestions of these bands and complete line lists can be found in the ESI. The fitting was done with PGOPHER.^34^ The numbers in parentheses give the uncertainty of the last digits

Parameter	Ground Hot 1	Hot 1	Ground Hot 2	Hot 2	Unit
*ν*		3234.6040(1)		3234.0608(1)	cm^−1^
*B*	4468.11(9)	4459.64(8)	4475.8(1)	4468.5(1)	MHz
*q*	3.27(6)^a^	3.27(6)^a^	<0.3^b^	<0.3^b^	MHz

a Kept equal for upper and lower state in the fit.

b ℓ-Type doubling not resolved for this band.

Our ground state rotational constant is in good agreement with those of Cernicharo *et al.*,^28^ but the precision has been increased by a factor of five. The band origin for *ν*_1_ was found to be 3237.132 cm^−1^, which is only 6 cm^−1^ higher than the best estimate value provided by Thorwirth *et al.*^27^

## 7 Conclusions and outlook

The spectroscopic experiments on the astrophysically relevant HC_3_O^+^ molecule have demonstrated that leak-out-spectroscopy, LOS, can be performed with excellent sensitivity and signal-to-noise ratio, permitting not only to detect the initially targeted *ν*_1_ band, but also some unexpected bands. In addition, we find that pulsed radiation sources can be employed for LOS measurements, as demonstrated in panel (a) of Fig. 1. This is especially interesting because pulsed OPO/OPA systems can be tuned over much wider frequency ranges than cw infrared sources. Even wider tuning is available for infrared free electron lasers, such as FELIX.^35^ Therefore, LOS might proof very useful to record wide-band vibrational spectra of mass-selected, bare ions. However, it needs to be examined in future experiments which low-lying vibrational modes can still supply sufficient kinetic energy to result in a LOS signal. Moreover, rotational – ro-vibrational double resonance using LOS was demonstrated here for the first time. Using this approach, seven high-resolution rotational lines in the mm-wave regime have been detected in this work for HC_3_O^+^, and the rotational fingerprints of other astrophysically important cations are currently being investigated in the Cologne laboratories.^29^ As an additional bonus in these double resonance experiments, due to the connectivity of transitions (see insets in Fig. 2), the rotational signals obtained *via* the fundamental *ν*_1_ and the *ν*_2_ + *ν*_5_ + *ν*_7_ combination band prove that these transitions are connected to the ground vibrational state, and are thus not hot bands.

In the present work, Ne gas has been used as neutral collision partner for LOS, with an ion-to-neutral mass ratio of 53 : 20 (=2.65). Experiments down to a temperature of about 12 K can be performed with Ne present in the trap in a constant fashion, and further cooling to nominally 4 K is possible by pulsing in a helium-diluted rare gas mixture into the trap. For obtaining more favorable mass ratios for LOS, in particular for the investigation of complex, heavier ions, higher-mass neutral partners, such as Ar, N_2_, Kr, Xe (with main isotopes ^40^Ar, ^14^N_2_, ^84^Kr, ^132^Xe) have to be chosen, of which the first two have been successfully tested in the course of this work and that of Schmid *et al.*^24^ Using Xe, we can thus imagine to investigate ions with masses as high as 132 × 2.65 = 350, or even beyond. Of course, these heavy rare gases come with the disadvantage of higher freeze-out temperatures (about 40 K for Ar and N_2_ in our experiments) and associated ion heating effect, but which can be circumvented using a pulsed rare gas mixture as mentioned above.

The observation of two hot vibrational bands in this work and one in our former work^24^ is surprising, given the cold trap temperatures. As LOS uses buffer gas collision partners heavier than Helium (Ne, N_2_ or Ar in our recent experiments), we suspect the collisions with these heavy species in the RF field to lead to heating of the stored ions. In fact, numerical simulations along these lines demonstrated this effect (see Fig. 7b in Asvany and Schlemmer^36^). We thus assume that the hot bending vibrations are not properly cooled and/or even excited in the Ne bath. Potentially, there might also be a detection bias due to favourable kick-out of ions performing bending vibrations (due to sterical arguments). An alternative scenario, where collisions of laser-excited HC_3_O^+^ with Ne atoms do not leave the ions in a completely quenched state, but in a vibrationally excited bending state, cannot serve as an explanation for the hot bands. The excitation from the ground state into, *e.g.*, the *ν*_1_ state and the excitation from a bending mode into the corresponding hot band are energetically different and spectroscopically well separated with our high-resolution laser and do not occur simultaneously in one trapping cycle. Further investigations along these lines can be used to obtain a more detailed understanding of the underlying vibration to translation (V–T) energy transfer process and to optimize the LOS signal.

**Fig. 3:**
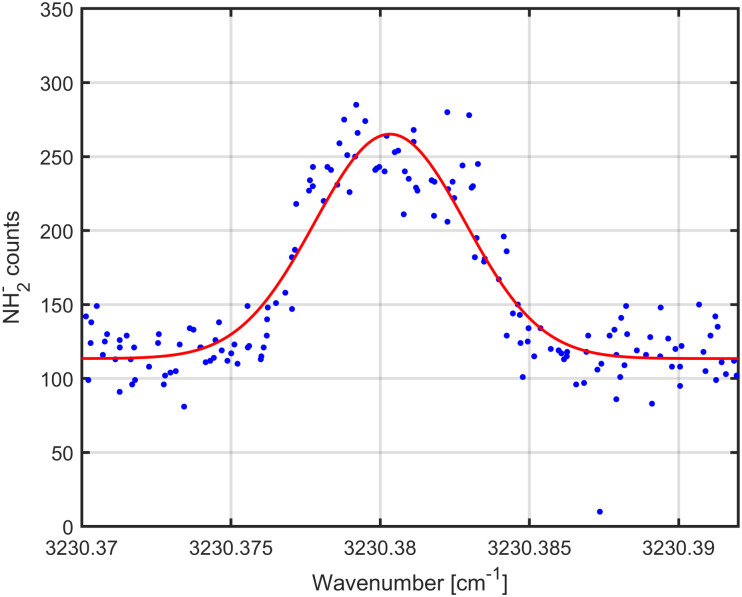
Transition *J*_KaKc_ = 2_02_ ← 1_01_ of the *ν*_3_ vibrational band of NH_2_^−^ measured with LOS.

There are several important advantages of LOS over other action spectroscopic methods, the most important one being its very general applicability to any cation or anion. In particular the application to anions is important as corresponding action spectroscopic methods were very limited to date.^10,21,22^ A first demonstration of the application of LOS to an anion is given in Fig. 3, depicting the *v*_3_ = 1 ← 0 *J*_*KaKc*_ = 2_02_ ← 1_01_ transition^37^ of NH_2_^−^. Up to date, LOS has been successfully tested on the anions NH_2_^−^, OH^−^, and HOCO^−^, simple cations such as CCCH^+^,^24^ C_2_H_2_^+^, HCO^+^, H_3_O^+^, NCCO^+^, H_2_CCCH^+^ and c-C_3_H_2_D^+^,^29^ but also on more complex and floppy systems such as C_2_H_3_^+^, CH_5_^+^, H_5_O_2_^+^, and CH_3_OH_2_^+^. It is thus even more generally applicable than the LIICG method (Laser Induced Inhibition of Complex Growth^11–13,20,38–40^), as that method relies on the attachment of helium atoms to the ion at 4 K, which may be very inefficient for some species, in particular for anions. A comparison of the two methods, LIICG *versus* LOS, is shown for the *ν*_1_*P*(6) line of HC_3_O^+^ in the ESI.† The LOS scheme, on the other hand, is based on the detection of the primary ions which often can be formed in sizable numbers while cation-helium complexes are only formed at low temperature from these primary ions in much smaller quantity. LOS is therefore very efficient and insensitive to small temperature changes. Furthermore, short trapping cycles of (*e.g.* less than 250 ms) per data point can be realized for LOS using powerful lasers. This allows to increase the overall duty cycle of the measurement, and thus to scan a complete spectrum in less time. This has not been realized in the current experiment operating at 4 K (we used trapping cycles of 1 s locked to our cold head), but in our previous work.^24^ Finally, the LOS scheme may be even extended to lower energy vibrational modes (<∼2000 cm^−1^), preferentially using a heavy neutral collision partner in order that the excited ion possesses enough energy to overcome the trap barrier. Corresponding experiments investigating the *ν*_3_ C–C stretching mode of HC_3_O^+^, using N_2_ as collision partner, are currently underway in our laboratory.

## Author contributions

Oskar Asvany: conceptualization, funding acquisition, validation, visualization, methodology, supervision, writing – original draft, writing – review & editing. Sven Thorwirth: investigation, – formal analysis, writing – review & editing. Philipp C. Schmid: investigation, writing – review & editing. Thomas Salomon: methodology, investigation, formal analysis, validation, writing – review & editing. Stephan Schlemmer: conceptualization, methodology, funding acquisition, writing – review & editing.

## Conflicts of interest

There are no conflicts of interest to declare.

## Supplementary Material

CP-025-D3CP01976D-s001
