# Supplementary material for: High-resolution ro-vibrational and rotational spectroscopy of HC3O+
Source: Phys Chem Chem Phys. 2023 Jul 4;25(29):19740–9. doi: 10.1039/d3cp01976d (PMC10370459; doi:10.1039/d3cp01976d)
Supplement: CP-025-D3CP01976D-s001 [file CP-025-D3CP01976D-s001.pdf]

Electronic Supplementary Material (ESI) for Physical Chemistry Chemical Physics.
This journal is © the Owner Societies 2023

Supplementary Material: High-resolution rovibrational and rotational spectroscopy of $HCCCO^+$

Oskar Asvany,* Sven Thorwirth, Philipp C. Schmid, Thomas Salomon, and Stephan Schlemmer

I. Physikalisches Institut der Universität zu Köln, Köln, Germany

E-mail: asvany@ph1.uni-koeln.de

1 Frequency calibration

The high-resolution results shown in Fig. 1 of the main paper have been recorded in sections of about 1.5 cm^{-1}, the scanning range of the OPO. Within such mode-hop-free sections, the precision of the measurement is a few 0.0001 cm^{-1}, but the total accuracy is only on the order of 0.001 cm^{-1}(see also comments below). For illustration purposes in that Figure, such sections have been combined with the help of overlapping prominent spectral features (the applied shifts are typically much less than 0.001 cm^{-1}), whereas for the analysis of the data the untouched measurements have been used.

Concerning the accuracy of our high-resolution data, it became clear during the analysis that the alignment of our wavemeter had not been optimal during the measurements and therefore showed slightly lower wavenumber readings. Therefore, the spectroscopic data were recalibrated using the 3204.730595 cm^{-1} line of neutral C_2H_2 contained within an absorption cell. Using this reference line (taken from the HITRAN database[1]), we shifted our original data up by 0.009 cm^{-1}. With this, we assume the accuracy of the data given in the Tables to be a few 0.001 cm^{-1}. The precision of the data, however, is much better than this, on the order of a few 0.0001 cm^{-1}.

2 Early ro-vibrational measurements applying LIICG

In summer 2020 we did first rovibrational measurements on $HCCCO^+$ using the LIICG method.[2] With the introduction of LOS, we continued the measurements using this novel method. The signal-to-noise ratio of LOS is superior as seen in the Figure below.

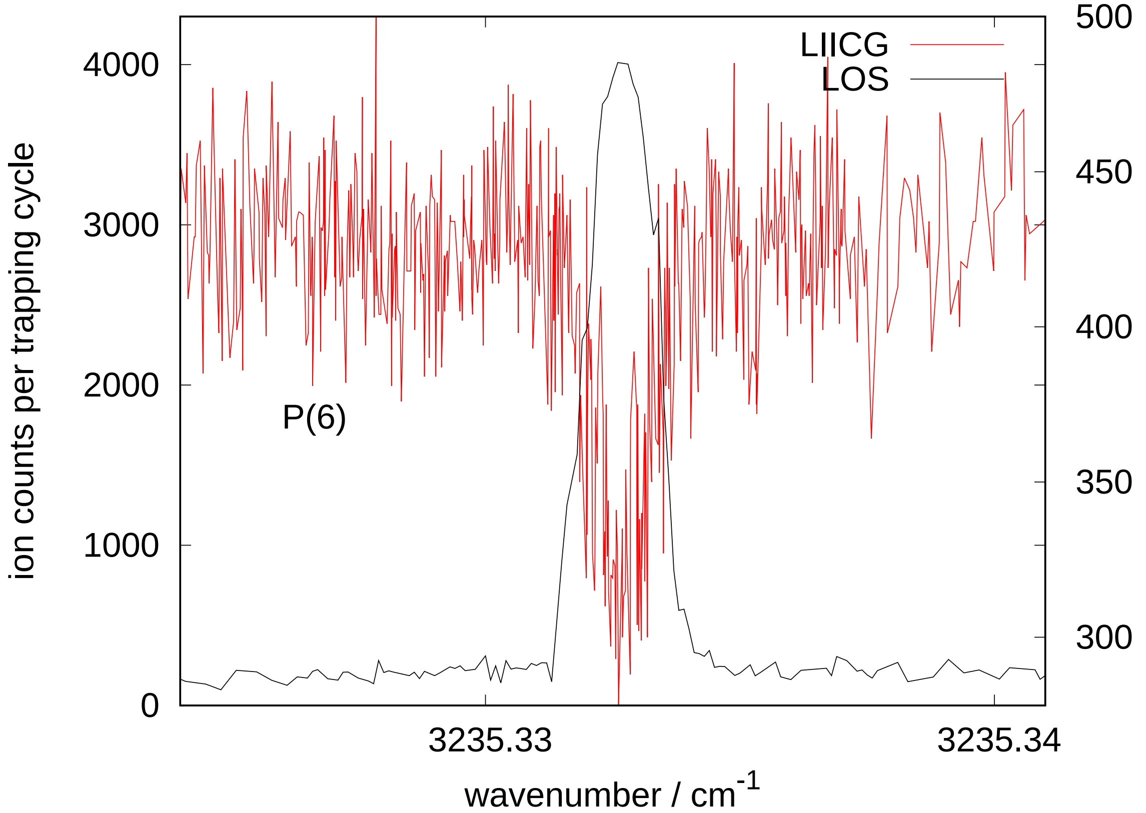

Figure S1: Comparison of measurements of the ν_1 $P(6)$ line of $HCCCO^+$ using LOS and LIICG. For LIICG, $HCCCO^+$-He complexes are generated at 4 K in the ion trap, which are finally counted (on the order of 450, see y2-axis). If the laser is in resonance with the $P(6)$ line of $HCCCO^+$, the counts of $HCCCO^+$-He decrease (dip at resonance). For LOS, on the other hand, one simply counts the $HCCCO^+$ ions which are kicked out of the ion trap at resonance (about 4000, see y1-axis). As visible, the S/N ratio of LOS is superior.

3 Comparison with earlier detection of ν_1

The Figure below shows a comparison of the low-resolution measurements of ν_1 and $\nu_2 + \nu_4$ of this work (Fig. 1a) with a former measurement of tagged Ne-$HCCCO^+$ published by Thorwirth et al.[3]. Both measurements exhibit two band structures, but the Ne-tagged measurement (red trace in Fig. S2) is blue-shifted by about 11 cm^{-1}. As the measurements in this work are corroborated by the well-calibrated high-resolution measurements (see Fig.1 b and c), and a blueshift of 11 cm^{-1} caused by the Ne-tag seems highly unlikely, we suspect a calibration error in measurement of the red trace. Together with theoretical predictions, this formerly mislead us to assign the left red peak to ν_1 and only speculate about the nature of the right peak, so that the $\nu_2 + \nu_4$ band could not be assigned with confidence in that work.[3]

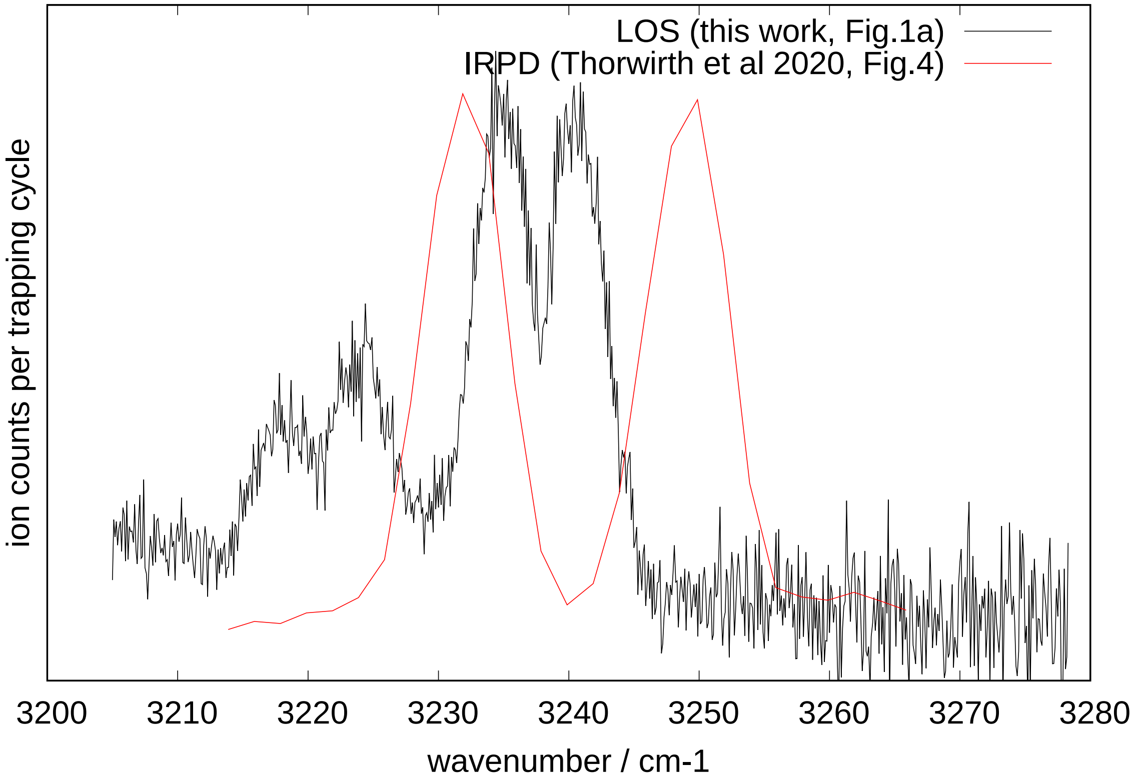

Figure S2: Comparison of the measurement of ν_1 and $\nu_2 + \nu_4$ of $HCCCO^+$ (this work, Fig. 1a) with infrared photodissociation (IRPD) of Ne-$HCCCO^+$ performed by Thorwirth et al.[3].

4 Resonance System

The rotational constants listed in Table 6 of the main manuscript yield experimental rotation-vibration interaction constants (α_1=13.63(4) MHz, α_{2+4}=23.07(5) MHz, α_{2+5+7}=11.97(5) MHz) significantly deviating from those estimated from the calculation (see Table 1 main manuscript; α_1=7.31 MHz, $\alpha_{2+4} \approx \alpha_2 + \alpha_4$=31.78 MHz, $\alpha_{2+5+7} \approx \alpha_2 + \alpha_5 + \alpha_7$=3.42 MHz). Although the presence of the $\nu_2 + \nu_4$ combination band might have been speculated (cf., Ref.[3]), the appearance of a third band in close proximity to ν_1 came somewhat as a surprise. A possible explanation for this finding is the existence of a (three-level) resonance system in which an initially dark state gains intensity from its resonance partners. In our case most likely a Fermi-resonance system is observed as the ν_1 fundamental as well as the ν_2+ν_4 combination band are both of Σ^+-symmetry. Hence, the initially dark state of the Fermi-resonance system has to posses at least one component of Σ^+-symmetry in order to borrow intensity from the bright states. This criterion is in general fulfilled by bands such as the $\nu_2 + \nu_5 + \nu_7$ combination band (Σ^+, Σ^- and Δ-symmetry) due to the two involved bending motions.

Within a simple second order de-perturbation approach all appearing coupling terms between the individual resonating bands should be equal in magnitude but opposite in sign ($\frac{|\langle\psi_i|W_{ij}|\psi_j\rangle|^2}{E_i-E_j} = -\frac{|\langle\psi_j|W_{ji}|\psi_i\rangle|^2}{E_j-E_i}$). Hence, the sum of their perturbed term values (Table S3 in the Supplementary Material), initially calculated from combination differences of the observed transitions, should be equal to the sum of their unperturbed term energies. An effective fit of the combined term values yields a rotational constant of the mixed states $B_{\text{eff}} = \frac{B_a+B_b+B_c}{3} = 4444.54(19)$ MHz which corresponds to an effective rotation-vibration interaction parameter $\alpha_{\text{eff}} = \frac{\alpha_a+\alpha_b+\alpha_c}{3} = 16.05(19)$ MHz. Taking the calculated rotation-vibration interaction constants of ν_1 and the $\nu_2 + \nu_4$ combination band as given above, the initially dark state of the resonance system should exhibit a rotation-vibration interaction constant $\alpha_{dark} = 9.1(6)$ MHz. A reasonable estimate for the rotation-vibration coupling

constant $\alpha_{\nu_2+\nu_5+\nu_7,l=0}$ of its two $\ell = 0$ (Σ^+- and Σ^--symmetry) components can be estimated from the values given in Table 1 in the main manuscript. The sum of the rotation-vibration coupling constants of the involved fundamentals has to be raised and lowered by the difference between the ℓ-type-doubling constants of the two bending motions yielding $\alpha_{\nu_2+\nu_5+\nu_7,\ell=0} = 3.42 \pm 5.26 = 8.68/-1.84$ MHz. The first value lies fairly good within the uncertainties of the derived dark state rotation-vibration coupling constant $\alpha_{dark} = 9.1(6)$ MHz supporting our tentative assignment.

5 Line lists and tentative assignment of hot bands of $HCCCO^+$

Two hot bands are observed in panel b of Fig. 1 in the main paper, an intense one with band center at about 3234.60 cm^{-1} (marked green in the Figure and called "hot1" in the following), and one less intense, with a lower band center at about 3234.06 cm^{-1} (marked blue in the Figure and called "hot2" in the following). We suspect them to originate from the lowest bending modes[3] of $HCCCO^+$, the C-C-C bending vibration ν_7 at 169[1]/174[2] cm^{-1}, its overtone $2\nu_7$, or the C-C-O bending vibration ν_6 at 558[1]/553[2] cm^{-1} (c.f. Table S1). We base our tentative assignments on characteristic observables such as band intensities, rotational constants B_ν (or rotation-vibration constants α_ν), doubling constants q_ν, and band centers (or anharmonicity constants χ).

The line lists of these two hot bands are given below. Due to the congested character of the Q-bands, only well-separated lines are listed here. The given rotational assignment is utilized to derive the term energies of the involved states by calculating combination differences. The hereby obtained ground state term values are listed in Tables S4 and S5, respectively, while those in the upper state of the hot bands are given in Table S6. The rotational constants listed in the mentioned tables refer to a least squares fit of the term values to a standard rigid rotor Hamiltonian neglecting centrifugal distortion.

[1]fc-CCSD(T)/aug-cc-pV(T+d)Z level of theory
[2]fc-CCSD(T)/ANO1 level of theory

A comparison of the obtained values with the results of the quantum chemical calculations given in Table S1 leaves room for two different assignments of the involved vibrational motions. One possible assignment would be "hot1" to originate from the C-C-O bending motion ν_6 ($^1\Pi$), while "hot2" originates from the C-C-C bending vibration ν_7 ($^1\Pi$). The major argument for this assignment is that their rotational-vibrational coupling constants α_ν agree in magnitude and sign with their predictions. Furthermore, the assigned $\nu_1 + \nu_6 \leftarrow \nu_6$ ($^1\Pi \leftarrow ^1 \Pi$) band would originate above the $\nu_1 + \nu_7 \leftarrow \nu_7$ ($^1\Pi \leftarrow ^1 \Pi$) band with a relative displacement (0.545 cm^{-1}) which is comparably close to the difference between the calculated anharmonicity constants χ of these two hot-bands (0.76 cm^{-1}, see Table S1). Also, a close inspection of "hot1" reveals resolved ℓ-type splittings for higher rotational states (see inset in Fig. 1b) which correspond to an ℓ-type doubling constant q_{hot1}= 3.26(5) MHz. This value is again reasonably close to the value q_6=3.06 MHz predicted for ν_6. On the other side, ℓ-type doubling is not observed for "hot2" which is somewhat surprising, as the l-type coupling constant of the C-C-C bending vibration, q_7, is calculated to be more than twice than the one of the C-C-O bending q_6.

However, taking into account our cryogenic experimental conditions, the assignment given above is difficult to accept, as we do not expect the ν_6 bending mode (at 558 cm^{-1} or about 803 K) to be significantly populated at T = 12 K. Therefore, we alternatively assign the more intense band "hot1" to $\nu_1 + \nu_7 \leftarrow \nu_7$ and the weaker band "hot2" to $\nu_1 + 2\nu_7 \leftarrow 2\nu_7$. Apart from this intensity argument, this assignment is corroborated by quantum levels in "hot2" apparently only existing for $J \geq 2$, which suggest an assignment to a $^1\Delta \leftarrow ^1 \Delta$ transition (but in this case we wonder why $2\nu_7$ appears as Δ-state (ℓ = 2) with a missing Σ-state (ℓ = 0)). Finally, we note that $\alpha_{hot2} \approx -15.96$ has double the value of $\alpha_{hot1} \approx -7.88$, as expected for an overtone state.

In summary, we can only tentatively assign the two hot bands to $\nu_1 + \nu_7 \leftarrow \nu_7$, $\nu_1 + 2\nu_7 \leftarrow 2\nu_7$, or $\nu_1 + \nu_6 \leftarrow \nu_6$. As an additional puzzle, both suggested assignment sets given above lack an explanation of the missing ℓ-type doubling in "hot2". This requires additional future

investigations of the bending vibrations to resolve the open questions.

line list of hot-band "hot1" of $HCCCO^+$

Truncated Log file from PGOPHER output. Transitions are given in units of cm^{-1}.

J'S'	#'	J"S"	#"	Observed	Calculated	Obs-Calc
13 e	1	14 e	1	3230.3785	3230.3780	0.0005
13 f	1	14 f	1	3230.3812	3230.3810	0.0002
12 f	1	13 f	1	3230.6862	3230.6863	-0.0001
11 e	1	12 e	1	3230.9886	3230.9885	0.0001
11 f	1	12 f	1	3230.9908	3230.9911	-0.0003
10 e	1	11 e	1	3231.2928	3231.2929	-0.0001
10 f	1	11 f	1	3231.2946	3231.2953	-0.0007
9 e	1	10 e	1	3231.5967	3231.5967	-0.0000
9 f	1	10 f	1	3231.5984	3231.5989	-0.0005
8 e	1	9 e	1	3231.8998	3231.9000	-0.0002
8 f	1	9 f	1	3231.9016	3231.9019	-0.0003
7 e	1	8 e	1	3232.2027	3232.2027	0.0000
7 f	1	8 f	1	3232.2041	3232.2044	-0.0003
6 e	1	7 e	1	3232.5050	3232.5048	0.0002
6 f	1	7 f	1	3232.5063	3232.5064	-0.0001
5 e	1	6 e	1	3232.8072	3232.8064	0.0008
5 f	1	6 f	1	3232.8072	3232.8077	-0.0005
				Blend	3232.8071	0.0001
4 f	1	5 f	1	3233.1081	3233.1085	-0.0004
4 e	1	5 e	1	3233.1081	3233.1074	0.0007
				Blend	3233.1080	0.0001
3 f	1	4 f	1	3233.4086	3233.4087	-0.0001
3 e	1	4 e	1	3233.4086	3233.4079	0.0007
				Blend	3233.4083	0.0003
2 e	1	3 e	1	3233.7085	3233.7078	0.0007
2 f	1	3 f	1	3233.7085	3233.7084	0.0001
				Blend	3233.7081	0.0004
1 f	1	2 f	1	3234.0077	3234.0075	0.0002
1 e	1	2 e	1	3234.0077	3234.0071	0.0006
				Blend	3234.0073	0.0004
10 f	1	10 e	1	3234.5608	3234.5610	-0.0002
9 f	1	9 e	1	3234.5688	3234.5688	0.0000
8 f	1	8 e	1	3234.5760	3234.5758	0.0002

7	f	1	7	e	1	3234.5822	3234.5821	0.0001
6	f	1	6	e	1	3234.5877	3234.5876	0.0001
4	f	1	4	e	1	3234.5962	3234.5962	0.0000
3	f	1	2	f	1	3235.4953	3235.4945	0.0008
3	e	1	2	e	1	3235.4953	3235.4952	0.0001
						Blend	3235.4949	0.0004
4	f	1	3	f	1	3235.7907	3235.7903	0.0004
4	e	1	3	e	1	3235.7907	3235.7911	-0.0004
						Blend	3235.7907	0.0000
5	f	1	4	f	1	3236.0859	3236.0854	0.0005
5	e	1	4	e	1	3236.0859	3236.0865	-0.0006
						Blend	3236.0859	-0.0000
6	e	1	5	e	1	3236.3806	3236.3813	-0.0007
6	f	1	5	f	1	3236.3806	3236.3800	0.0006
						Blend	3236.3806	-0.0000
7	f	1	6	f	1	3236.6751	3236.6740	0.0011
						Blend	3236.6748	0.0003
8	f	1	7	f	1	3236.9676	3236.9675	0.0001
8	e	1	7	e	1	3236.9695	3236.9692	0.0003
9	f	1	8	f	1	3237.2606	3237.2603	0.0003
9	e	1	8	e	1	3237.2625	3237.2623	0.0002
10	f	1	9	f	1	3237.5520	3237.5527	-0.0007
10	e	1	9	e	1	3237.5543	3237.5548	-0.0005
11	f	1	10	f	1	3237.8436	3237.8444	-0.0008
11	e	1	10	e	1	3237.8462	3237.8468	-0.0006
12	f	1	11	f	1	3238.1352	3238.1356	-0.0004
12	e	1	11	e	1	3238.1381	3238.1382	-0.0001
13	f	1	12	f	1	3238.4262	3238.4262	-0.0000
13	e	1	12	e	1	3238.4294	3238.4291	0.0003
14	f	1	13	f	1	3238.7159	3238.7163	-0.0004
14	e	1	13	e	1	3238.7191	3238.7194	-0.0003
15	f	1	14	f	1	3239.0057	3239.0058	-0.0001
15	e	1	14	e	1	3239.0095	3239.0091	0.0004
16	f	1	15	f	1	3239.2950	3239.2947	0.0003
16	e	1	15	e	1	3239.2990	3239.2982	0.0008
17	f	1	16	f	1	3239.5829	3239.5831	-0.0002
17	e	1	16	e	1	3239.5872	3239.5868	0.0004

62 observations in total reduced after combining blends to 52

line list of hot-band "hot2" of $HCCCO^+$

Truncated Log file from PGOPHER output. Transitions are given in units of cm^{-1}. All Λ-doubled lines are blended (there are 2×22 lines in the list).

J'S'	#'	J"S"	#"	Observed	Calculated	Obs-Calc
10 f	1	11 f	1	3230.7501	3230.7495	0.0006
10 e	1	11 e	1	3230.7501	3230.7495	0.0006
9 f	1	10 f	1	3231.0529	3231.0529	-0.0001
9 e	1	10 e	1	3231.0529	3231.0529	-0.0001
8 f	1	9 f	1	3231.3556	3231.3559	-0.0003
8 e	1	9 e	1	3231.3556	3231.3559	-0.0003
7 f	1	8 f	1	3231.6585	3231.6584	0.0001
7 e	1	8 e	1	3231.6585	3231.6584	0.0001
6 e	1	7 e	1	3231.9602	3231.9604	-0.0002
6 f	1	7 f	1	3231.9602	3231.9604	-0.0002
4 f	1	5 f	1	3232.5630	3232.5630	0.0000
4 e	1	5 e	1	3232.5630	3232.5630	0.0000
3 f	1	4 f	1	3232.8636	3232.8635	0.0001
3 e	1	4 e	1	3232.8636	3232.8635	0.0001
2 f	1	3 f	1	3233.1634	3233.1636	-0.0002
2 e	1	3 e	1	3233.1634	3233.1636	-0.0002
8 f	1	8 e	1	3234.0430	3234.0433	-0.0003
8 e	1	8 f	1	3234.0430	3234.0433	-0.0003
7 e	1	7 f	1	3234.0472	3234.0472	-0.0000
7 f	1	7 e	1	3234.0472	3234.0472	-0.0000
6 f	1	6 e	1	3234.0508	3234.0506	0.0002
6 e	1	6 f	1	3234.0508	3234.0506	0.0002
5 f	1	5 e	1	3234.0531	3234.0535	-0.0004
5 e	1	5 f	1	3234.0531	3234.0535	-0.0004
3 f	1	2 f	1	3234.9536	3234.9537	-0.0001
3 e	1	2 e	1	3234.9536	3234.9537	-0.0001
4 e	1	3 e	1	3235.2504	3235.2503	0.0001
4 f	1	3 f	1	3235.2504	3235.2503	0.0001
5 e	1	4 e	1	3235.5467	3235.5465	0.0002
5 f	1	4 f	1	3235.5467	3235.5465	0.0002
6 f	1	5 f	1	3235.8419	3235.8422	-0.0003
6 e	1	5 e	1	3235.8419	3235.8422	-0.0003
7 f	1	6 f	1	3236.1375	3236.1374	0.0001
7 e	1	6 e	1	3236.1375	3236.1374	0.0001
8 e	1	7 e	1	3236.4325	3236.4321	0.0005

8	f	1	7	f	1	3236.4325	3236.4321	0.0005
9	e	1	8	e	1	3236.7268	3236.7263	0.0005
9	f	1	8	f	1	3236.7268	3236.7263	0.0005
10	e	1	9	e	1	3237.0207	3237.0200	0.0006
10	f	1	9	f	1	3237.0207	3237.0200	0.0006
11	e	1	10	e	1	3237.3123	3237.3132	-0.0009
11	f	1	10	f	1	3237.3123	3237.3132	-0.0009
12	e	1	11	e	1	3237.6058	3237.6060	-0.0002
12	f	1	11	f	1	3237.6058	3237.6060	-0.0002

6 Quantum chemical calculations

Table S1: Fundamental vibrational wavenumbers of HC_3O^+, their rotation-vibration coupling constants α_ν, and doubling constants q_ν, calculated on the fc-CCSD(T)/pVTZ level of theory (this work). The given anharmonicity constants $\chi_{1\nu}$ refer to the hot-bands of the C-H stretching fundamental ν_1.

Mode	Harm [cm^{-1}]	Anharm [cm^{-1}]	$v^a_{i,BE}$ [cm^{-1}]	α_i [MHz]	q_i [MHz]	χ_{1i} ^{b} [cm^{-1}]
$\nu_1(\Sigma)$ C-H stretch	3362.1	3228.6	3231	7.31	-	-54.52
$\nu_2(\Sigma)$ C-O stretch	2353.1	2312.7	2316	22.84	-	-1.37
$\nu_3(\Sigma)$ C-C stretch	2097.4	2063.6	2074	14.56	-	-7.86
$\nu_4(\Sigma)$ C-C stretch	917.8	901.3	911	8.94	-	1.14
$\nu_5(\Pi)$ C-C-H bending	773.2	757.5	773	-0.49	2.13	-18.56
$\nu_6(\Pi)$ C-C-O bending	552.3	547.3	558	-8.00	3.06	-0.39
$\nu_7(\Pi)$ C-C-C bending	181.1	166.1	169	-18.93	7.39	-1.49

^{a} Best estimate value, see Thorwirth et al.[3]

^{b} anharmonicity constants of the hot-bands with respect to the ν_1 C-H stretching fundamental

7 Ground state term values

Table S2: Term values for the vibrational ground state ($\nu = 0$) of $HCCCO^+$ as derived from all observed transitions of the ν_1 C-H stretching fundamental and resonating combination bands ($\nu_2 + \nu_4$, $\nu_2 + \nu_5 + \nu_7$). Two separate networks of states are derived from the experimental data, denoted as a and b. This leads to two reference points $E\text{-}D_0 = 0$ that are not directly linked to each other. These sets are combined based on their y-axis intercept assuming the validity of the rigid rotor approximation.

J	Set	$E\text{-}D_0^i$ [cm^{-1}]	$E\text{-}D_0^{ii}$ [cm^{-1}]	$E_{combined}$ [cm^{-1}]
0	a	0	-	0.00000
1	b	-	0	0.29760(1)
2	a	0.89282(16)	-	0.89282(16)
3	b	-	1.48835(3)	1.78595(2)
4	a	2.97627(9)	-	2.97627(9)
5	b	-	4.16701(9)	4.46461(10)
6	a	6.25001(12)	-	6.25001(12)
7	b	-	8.03588(8)	8.33349(9)
8	a	10.71430(21)	-	10.71430(21)
9	b	-	13.09521(18)	13.39281(20)
10	a	16.36868(48)	-	16.36868(48)
11	b	-	19.34491(67)	19.64251(68)
12	a	23.21350(52)	-	23.21350(52)
13	b	-	26.78503(96)	27.08263(97)
14	a	31.24868(94	-	31.24868(94)
15	b	-	35.41525(105)	35.71286(107)
16	a	40.47311(260)	-	40.47311(260)
**B_0 [MHz]**		4460.90(5)	4461.00(3)	4460.94(3)

8 Term values of C-H stretching fundamental and resonating combination bands

Table S3: Term energies for the observed vibrationally excited states lying in the C-H-stretching range of $HCCCO^+$. The given energies are calculated from the observed transitions utilizing the combined ground state term values given in Table S2.

**J**	ν_1**=1 [cm^{-1}]**	ν_2**=1,**ν_4**=1 [cm^{-1}]**	ν_2**=1,**ν_5**=1,**ν_7**=1 [cm^{-1}]**
0	3237.13231(1)	3220.46705(1)	-
1	3237.42911(16)	3220.76302(16)	3237.59800(16)
2	3238.02246(2)	3221.35538(2)	3238.19314(2)
3	3238.91240(9)	3222.24355(9)	3239.08143(9)
4	3240.09956(10)	3223.42815(10)	3240.26938(10)
5	3241.58269(12)	3224.90820(12)	3241.75301(12)
6	3243.36323(9)	3226.68482(9)	3243.53421(9)
7	3245.43974(21)	3228.75722(21)	3245.61172(21)
8	3247.81359(20)	3231.12580(20)	3247.98621(20)
9	3250.48364(48)	3233.79005(48)	3250.65727(48)
10	3253.45050(68)	3236.75096(68)	3253.62520(68)
11	3256.71401(52)	3240.00706(52)	3256.88964(52)
12	3260.27453(97)	3243.56005(97)	3260.45164(97)
13	3264.13109(94)	3247.40882(94)	3264.30933(94)
14	3268.28504(107)	3251.56214(107)	3268.46464(107)
15	3272.73379(260)	3255.99306(260)	3272.91519(260)
16	3277.48248(107)	3260.72864(107)	3277.66526(107)
17	3282.52468(260)	3265.76015(260)	3282.70927(260)
**B_ν [MHz]**	4447.20(3)	4437.72(18)	4448.71(4)
α_ν^a **[MHz]**	13.74(5)	23.22(19)	12.23(6)
$\alpha_{\nu,calc}^b$ **[MHz]**	7.31	31.78	3.42

[a] $\alpha_\nu = B_0 - B_\nu$, B_0 is taken from the combined fit in Table S2.
[b] fc-CCSD(T)/pVTZ, this study

9 Term values derived from hot-bands

Table S4: Term values for the ground state of the first hot-band (Hot 1) of $HCCCO^+$ derived from ground state combination differences. Four separate networks of states are derived from the experimental data, denoted as a,b,c and d, due to the two l-type components of the underlying motion. This leads to four reference points E-D_0 = 0 that are not directly linked to each other. These sets are combined based on their y-axis intercept assuming the validity of the rigid rotor approximation.

$J_{e,f}$	Set	E-D_0^i [cm^{-1}]	E-D_0^{ii} [cm^{-1}]	$E_{combined}$ [cm^{-1}]
1_f	a	0.0000	-	0.0000
1_e	-	-	0.0000	0.0000
2_f	b	0.0000		0.5961(8)
2_e	d	-	0.0000	0.5961(4)
3_f	a	1.4908(6)	-	1.4908(6)
3_e	c	-	1.4908(6)	1.4908(5)
4_f	b	2.0870(6)	-	2.6830(10)
4_e	d	-	2.0870(6)	2.6831(7)
5_f	a	4.1742(8)		4.1742(8)
5_e	c	-	4.1742(8)	4.1742(10)
6_f	b	5.3658(8)	-	5.9619(11)
6_e	d	-	5.3670(8)	5.9631(9)
7_f	a	8.0492(10)	-	8.0492(10)
7_e	c	-	8.0504(10)	8.0504(11)
8_f	b	9.8373(10)	-	10.4335(12)
8_e	d	-	9.8402(10)	10.4363(10)
9_f	a	13.1162(11)	-	13.1162(11)
9_e	c	-	13.1210(11)	13.1210(12)
10_f	b	15.5000(11)	-	16.0980(14)
10_e	d	-	15.5066(11)	16.1027(12)
11_f	a	19.3745(13)	-	19.3745(13)
11_e	c	-	19.3836(13)	19.3836(14)
12_f	b	22.3537(13)	-	22.9498(15)
12_e	d	-	22.3653(13)	22.9614(13)
13_f	a	26.8246(14)	-	26.8246(14)
13_e	c	-	26.8391(14)	26.8391(15)
14_f	b	30.4000(14)	-	30.9960(16)
14_e	d	-	30.4173(14)	31.0134(14)
B_a [MHz]		4467.66(17)	B_b [MHz]	4467.46(15)
B_c [MHz]		4470.22(9)	B_d [MHz]	4470.09(5)
$B_{combined}$ [MHz]			4468.82(34)	
$\alpha^a_{combined}$ [MHz]			-7.88(34)	
$\alpha^b_{\nu_6=1,calc}$ [MHz]			-8.00	
$\alpha^b_{\nu_7=1,calc}$ [MHz]			-18.93	

[a] α_ν=B_0-B_ν, B_0 is taken from the combined fit in Table S2.
[b] fc-CCSD(T)/pVTZ, this study

Table S5: Term values for the ground state of the second hot-band (Hot 2) of $HCCCO^+$ as derived from ground state combination differences. Four separate networks of states are derived from the experimental data, denoted as a,b,c and d, due to the two l-type components of the underlying bending motion. This leads to four reference points E-$D_0 = 0$ that are not directly linked to each other. These sets are combined based on their y-axis intercept assuming the validity of the rigid rotor approximation.

$J_{e,f}$	Set	E-$D_0^{a,c}$ [cm^{-1}]	E-$D_0^{b,d}$ [cm^{-1}]	$E_{combined}$ [cm^{-1}]
1_e	-	-	-	0.000000
1_f	-	-	-	0.000000
2_e	c	0.000000	-	0.59733(1)
2_f	d	-	0.000000	0.59733(1)
3_e	a	0.000000	-	1.49336(3)
3_f	b	-	0.000000	1.49336(3)
4_e	c	2.09043(57)	-	2.68776(57)
4_f	d		2.09043(57)	2.68776(57)
5_e	a	2.68793(57)	-	4.18129(57)
5_f	b		2.68793(57)	4.18129(57)
6_e	c	5.37611(80)	-	5.97344(80)
6_f	d		5.37611(80)	5.97344(80)
7_e	a	6.57075(80)	-	8.06410(80)
7_f	b	-	6.57075(80)	8.06410(80)
8_e	c	9.85601(98)	-	10.45334(98)
8_f	d	-	9.85601(98)	10.45334(98)
9_e	a	11.64856(98)	-	13.14191(98)
9_f	b	-	11.64856(98)	13.14191(98)
10_e	c	15.53081(113)	-	16.12814(113)
10_f	d	-	15.53081(113)	16.12814(113)
11_e	a	17.92007(113)	-	19.41343(113)
11_f	b	-	17.92007(113)	19.41343(113)
12_e	c	22.39959(127)	-	22.99692(127)
12_f	d	-	22.39959(127)	22.99692(127)
B_a [MHz]		4476.97(8)	B_b [MHz]	4476.97(8)
B_c [MHz]		4476.86(5)	B_d [MHz]	4476.86(5)

$B_{combined}$ [MHz]	4476.90(3)
$\alpha^a_{combined}$ [MHz]	-15.96(5)
$\alpha^b_{\nu_7=1,calc}$ [MHz]	-18.93
$\alpha^b_{\nu_7=2,calc}$ [MHz]	-37.86

[a] α_ν=B_0-B_ν, B_0 is taken from the combined fit in Table S2.
[b] fc-CCSD(T)/pVTZ, this study

Table S6: Term values of the excited states of the observed hot-bands of $HCCCO^+$. The given energies are calculated from the observed transitions utilizing the combined ground state term values given in Tables S4 and S5. All units are given in wavenumbers unless otherwise noted.

J	Hot 2	Hot 1
1_e	3234.0591(4)	3234.6033(5)
1_f	3234.0591(4)	3234.6033(9)
2_e	3234.6562(4)	3235.1985(7)
2_f	3234.6562(4)	3235.1985(7)
3_e	3235.5508(7)	3236.0911(8)
3_f	3235.5508(7)	3236.0911(10)
4_e	3236.7437(7)	3237.2819(10)
4_f	3236.7437(7)	3237.2817(9)
5_e	3238.2344(9)	3238.7698(10)
5_f	3238.2344(9)	3238.7686(12)
6_e	3240.0237(9)	3240.5558(12)
6_f	3240.0237(9)	3240.5547(11)
7_e	3242.1111(11)	3242.6392(11)
7_f	3242.1111(11)	3242.6364(13)
8_e	3244.4969(11)	3245.0219(13)
8_f	3244.4969(11)	3245.0155(12)
9_e	3247.1804(12)	3247.7008(13)
9_f	3247.1804(12)	3247.6921(14)
10_e	3250.1629(12)	3250.6779(14)
10_f	3250.1629(12)	3250.6668(13)
11_e	3253.4424(13)	3253.9518(14)
11_f	3253.4424(13)	3253.9379(15)
12_e	3257.0205(12)	3257.5248(15)
12_f	3257.0205(12)	3257.5079(14)
13_e	3260.8946(13)	3261.3940(15)
13_f	3260.8946(13)	3261.3741(16)
14_e	-	3265.5588(15)
14_f	-	3265.5409(14)
15_e	-	3270.0233(15)
15_f	-	3270.0021(16)
**B_ν [MHz]**	4469.64(7)	4460.32(42)
**α_ν^a [MHz]**	7.26(8)	8.50(54)
**$\alpha_{\nu,calc}^b$ [MHz]**	7.31	7.31

[a] $\alpha_\nu = B_{\nu_{hot},\nu_2} - B_{\nu_{hot}}$, the values of $B_{\nu_{hot}}$ are taken from the combined fits in Table S4 and S5.

[b] fc-CCSD(T)/pVTZ, taken from ν_1 stretching fundamental

References

(1) Gordon, I.; Rothman, L.; Hargreaves, R.; Hashemi, R.; Karlovets, E.; Skinner, F.; Conway, E.; Hill, C.; Kochanov, R.; Tan, Y. et al. The HITRAN2020 molecular spectroscopic database. *Journal of Quantitative Spectroscopy and Radiative Transfer* **2022**, *277*, 107949.

(2) Asvany, O.; Brünken, S.; Kluge, L.; Schlemmer, S. COLTRAP: a 22-pole ion trapping machine for spectroscopy at 4 K. *Appl. Phys. B* **2014**, *114*, 203–211.

(3) Thorwirth, S.; Harding, M. E.; Asvany, O.; Brünken, S.; Jusko, P.; Lee, K. L. K.; Salomon, T.; McCarthy, M. C.; Schlemmer, S. Descendant of the X-ogen carrier and a 'mass of 69': infrared action spectroscopic detection of HC_3O^+ and HC_3S^+. *Molecular Physics* **2020**, *118*, e1776409.
